# Evaluating the effect of maternal mHealth text messages on uptake of maternal and child health care services in South Africa: a multicentre cohort intervention study

**DOI:** 10.1186/s12978-020-01017-3

**Published:** 2020-10-20

**Authors:** Jesse Coleman, Vivian Black, Anna Ekéus Thorson, Jaran Eriksen

**Affiliations:** 1 Wits Reproductive Health & HIV Institute, Faculty of Health Sciences, University of the Witwatersrand, 22 Esselen Street, Hillbrow, Johannesburg, 2000 South Africa; 2 Department of Global Public Health, Karolinska Institutet, 171 77 Stockholm, Sweden; 3 Department of Clinical Microbiology and Infectious Diseases, Faculty of Health Sciences, University of the Witwatersrand, Johannesburg, 1 Jan Smuts Avenue, Braamfontein, , Johannesburg, 2000 South Africa; 4 Department of Laboratory Medicine, Karolinska Institutet, C1 68, Karolinska University Hospital, Huddinge, 141 86 Stockholm, Sweden

**Keywords:** Maternal health, Newborn health, south africa, mHealth, SMS

## Abstract

**Background:**

There are high expectations that mobile health (mHealth) strategies will increase uptake of health care services, especially in resource strained settings. Our study aimed to evaluate effects of an mHealth intervention on uptake of maternal health services.

**Methods:**

This was an intervention cohort study conducted at six public antenatal and postnatal care clinics in inner-city Johannesburg, South Africa. The intervention consisted of twice-weekly informative and pregnancy stage-based maternal health information text messages sent to women during pregnancy until their child was one year of age. The intervention arm of 87 mother-infant pairs was compared to a control arm of 90 pairs. Univariate and multivariate analyses were used to compare the probability of the outcome between the two groups.

**Results:**

Intervention participants had higher odds of attending all government-recommended antenatal and postnatal visits, all recommended first year vaccinations (OR: 3.2, 95% CI 1.63–6.31) and had higher odds of attending at least the recommended four antenatal visits (OR: 3.21, 95% CI 1.73–5.98).

**Conclusion:**

We show an improvement in achieving complete maternal-infant continuum of care, providing evidence of a positive impact of informative maternal mHealth messages sent to pregnant women and new mothers.

*Trial registration* ISRCTN, ISRCTN41772986. Registered 13 February 2019—Retrospectively registered, https://www.isrctn.com/ISRCTN41772986

## Background

Attendance to professional maternal and infant health services during pregnancy (antenatal care or ANC) and postnatal care (PNC), including maternal and infant vaccinations, are key contributors to a healthy pregnancy, delivery and child [1]. Together, ANC, delivery with a skilled birth attendant, PNC and infant vaccinations constitute the core of the maternal, neonatal, and infant health continuum of care [2]. ANC visits allow medical professionals to identify health problems related to the pregnancy [3]. It has been found that women attending more ANC visits have lower perinatal morbidity and mortality than those who have fewer antenatal care visits, with a more pronounced effect in low-and middle income countries [4, 5]. The World Health Organization (WHO) currently recommends a minimum of four visits for all pregnancies [6].

Starting with the introduction of the smallpox vaccine in 1796, vaccines have revolutionised global public health. The 2016 World Health Organisation Expanded Programme on Immunization (EPI) guidelines recommend infant immunization start within 24 h of birth and continue along a planned schedule until all recommended doses are received [7]. Globally, EPI programmes have been successful and immunization rates in the first year of life are high; rates for diphtheria, tetanus and pertussis (DTP) and polio immunization stood at 85% in 2019, with measles and Hepatitis B coverage at 85% and 85% respectively [8].

The use of mobile phones to improve health outcomes, strengthen health systems, or increase patient engagement with the health system, known as mobile health (mHealth), is relatively new, but holds promise [9, 10]. To date, patient-focused mHealth interventions have been shown to increase adherence to antiretroviral medication [11, 12], support patients with chronic diseases [13], and increase attendance to maternal and infant care services [14, 15]. Despite this, there are knowledge gaps related to the implementation of mHealth interventions among pregnant women in many settings and detailed information on the type and structure of effective mHealth interventions is incomplete.

ANC and PNC are not being utilised optimally in South Africa as has been shown by WHO immunization data for South Africa, studies looking at South African EPI coverage, measles outbreaks, and poor treatment of maternal health patients [16, 17]. Moreover, the high prevalence of HIV among women of reproductive age, recently estimated at 22.3% [18], warrants close follow-up of HIV-positive pregnant women throughout the maternal health continuum of care to identify high-risk pregnancies, to prevent mother-to-child transmission of HIV (PMTCT) and to ensure antiretroviral treatment access to the infected woman [19, 20]. An effective maternal health intervention could improve maternal and infant health. Maternal mHealth interventions have been identified as potential tools to support the continuum in resource strained settings [14, 21]. This study assessed whether a maternal mHealth intervention, using informational maternal health SMSes timed to the stage of pregnancy and age of infant, was an effective strategy to increase rates of ANC attendance, EPI coverage, and comprehensive maternal, neonatal and infant care in inner-city Johannesburg, South Africa.

## Methods

### Study setting

This multi-centre cohort intervention study ran from May 2014 to June 2015 and included maternal-infant pairs recruited from six participating public healthcare facilities offering ANC and PNC/EPI services in the inner city of Johannesburg and Hillbrow, urban neighbourhoods within Johannesburg’s inner-city. These neighbouring areas have a high population density, are predominantly low-income and have high rates of alcohol abuse, gender-based violence, unemployment (estimated at 23% in 2013) and HIV (27% HIV positivity among pregnant women in 2013) [22, 23]. There are 16 public healthcare facilities offering ANC and PNC/EPI services in inner city Johannesburg, and Mobile Alliance for Maternal Action (MAMA) was offered in six of these. Among these six, three were selected as intervention facilities, and another three were selected as control facilities from the 10 not offering the MAMA intervention. All sites provided standard ANC and PNC services to study participants and were purposively selected based on client similarity and proximity to each other.

### Intervention

The intervention consisted of free one-way maternal health SMSes sent twice weekly throughout pregnancy and for one year postnatally. The SMSes, which contained supportive and informative information timed to the stage of pregnancy and age of the child, sent as part of the Mobile Alliance for Maternal Action (MAMA) South Africa project [24]. The SMS content was initially drafted by BabyCentre UK and then customised for the South African context by a team of local maternal and infant health professionals. The SMSes covered a range of maternal and infant health topics such as healthy eating, reminders to go for ANC/PNC appointments, psycho-social support, PMTCT support messages (if HIV-related messages were requested) and delivery planning (for examples, see Appendix). The intervention was offered to all pregnant women receiving ANC care at the intervention sites and supplemented the clinical standard of care offered. In this setting 98.4% of households owned a mobile phone [25] and almost all the study women had their own phone that they received the intervention messages on. Intervention participants joined the SMS intervention between their 11th and 39th week of pregnancy, thereby receiving between two and 28 intervention SMSes before delivery. An additional 104 messages were sent postnatally, and included reminders for each vaccination during the first year.

### Participants and sample size

Study recruitment was initiated two years after the SMSes were first offered. Intervention participants were identified from a list of the SMS recipients who had received the full year of postnatal messages. All women for whom the telephone number was listed, were contacted by phone and invited to participate. Each woman was called up to five times on separate days if there was no answer to the phone call. Control arm participants were identified while they were receiving PNC services at a control recruitment site, screened for eligibility and invited to participate.

All women within the mother–infant pairs in both the intervention and control groups were required to be over the age of 18 at recruitment, to have received ANC and PNC services at one the participating ANC/PNC sites between July 2012 and June 2014, to have delivered with a skilled birth attendant at one of two participating delivery sites, and to have had regular access to a cellular phone. All participants were also required to attend a face-to-face interview and provide their infant’s Road to Health (RTH) monitoring booklet.

The primary outcome was the proportion of mother–child pairs who would receive comprehensive maternal, neonatal and infant care. The aim was to include as many as possible of the women who had signed up for the intervention. Due to the low number of women who could be included, we made a post hoc sample size calculation. The sample size was based on complete EPI coverage at one year of age as no previous data for the composite score could be found. No reliable local data were available, so we used the 2013 WHO data on South Africa’s measles vaccination coverage rate at one year of age, which was 66%, as a baseline [26]. To identify an increase in coverage from 66 to 86%, the minimum required for herd immunity from most childhood vaccines [27], at 80% power and 95% confidence, a sample size of 68 individuals per arm was identified [28].

### Data collection and sampling

**Fig. 1:**
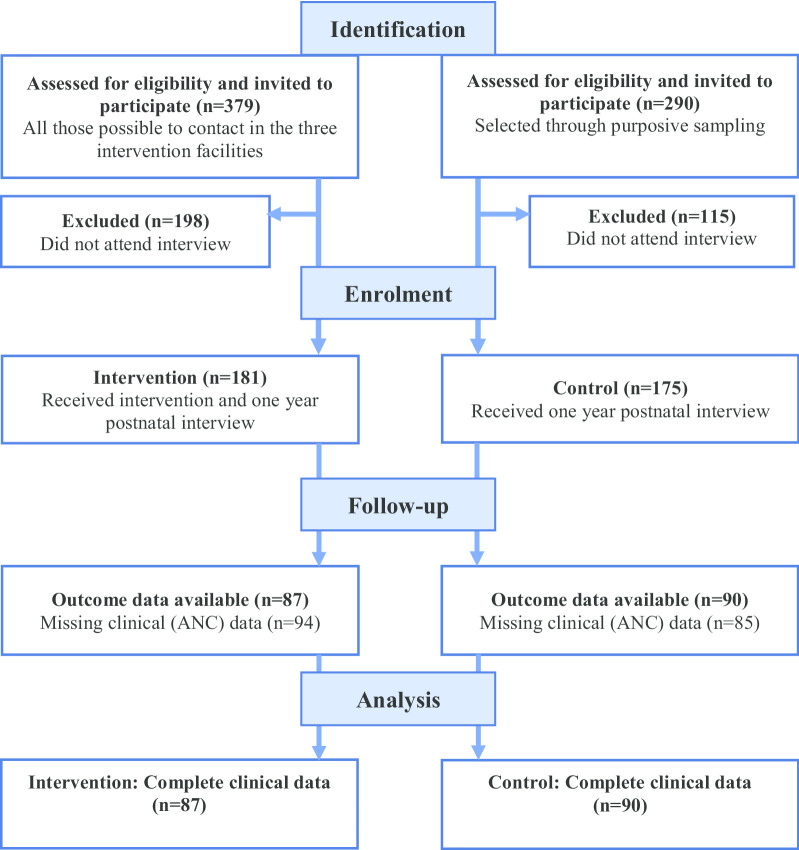
Participant flow diagram

Socio-demographic data were collected during participant interviews. ANC attendance data were collected from clinical ANC records and EPI coverage data were collected from infant RTH booklets. All study data were digitised and stored using Research Electronic Data Capture (REDCap), hosted at the University of Witwatersrand. REDCap is a secure, web-based application designed to support data capture for research studies [29]. Across the three intervention sites, 1770 women signed up to receive the SMSes. Of those, 379 (21.4%) could be reached by phone (for the rest there was no telephone number or the person did not respond to the phone call) and were invited to participate in the study of which 181 (47.8%) showed up for the interview. In the control arm, 290 participants were identified and invited to participate while 175 (60.3%) attended the interview. Just over half of the 356 women interviewed (n = 179, 50.3%) had missing ANC records and were excluded from the analysis (see Fig. 1). Complete data for all outcomes was available for a total of 177 individuals; 87 in the intervention arm and 90 in the control.

### Outcomes

Continuum of care is typically defined by the data available in individual studies [30, 31]. The study team defined the primary outcome as a binary composite of two maternal and infant indicators; having had four or more ANC visits (indicator 1), and receiving all first-year infant vaccinations at one year of age (indicator 2). These indicators are based on recommendations from the South African National Department of Health maternal health and EPI guidelines [32, 33]. Delivery with a skilled birth attendant, a common indicator within other maternal health continuum of care work [31], was not included as it was part of the study inclusion criteria. Notably, rates of facility-based births in South African urban settings are very high; recently estimated to be 99% in Gauteng province, where Johannesburg is located [34].

Secondary outcomes included attendance to at least two, three, four and five ANC visits, the mean number of ANC visits attended, and mean vaccination coverage of the two groups. The study aim was not to focus on HIV-related outcomes in part due to data on a related HIV-positive cohort having been published elsewhere [35].

### Analysis

Chi-square tests were conducted on binary variables, Student’s *t*-tests were used for continuous variables and univariate and multivariate analyses were used to compare the probability of the outcome between the two groups. Pre-existing socio-demographic cohort differences were identified and adjusted for in the model, as noted. All data analysis was conducted using Stata version 13 [36] and statistical significance was considered at p < 0.05.

## Results

In total, 87 mother–child pairs were included in the intervention group and 90 in the control. Both groups had similar socio-demographic and baseline clinical characteristics (Tables 1 and 2). However, a difference in country of birth was noted with 45% of the intervention group being born in South Africa compared to 29% in the control group (p = 0.004).

**Table 1:** Study population characteristics (proportions)

	Intervention n = 87	Control n = 90	Overall *p* value
Nationality			
South African	0.45 (0.26–0.65)	0.29 (0.23–0.36)	0.04
Zimbabwe	0.54 (0.37–0.70)	0.57 (0.50–0.63)	
Other nationality	0.01 (0.00–0.14)	0.14 (0.12–0.17)	
Highest level of education			
Completed secondary school or higher	0.55 (0.48–0.62)	0.52 (0.47–0.57)	0.40
Home language			
IsiNdebele	0.45 (0.30–0.61)	0.31 (0.16–0.52)	0.20
IsiZulu	0.26 (0.22–0.31)	0.28 (0.22–0.35)	
Other	0.29 (0.18–0.42)	0.41 (0.29–0.55)	
Employment status			
Employed full-time	0.24 (0.21–0.28)	0.33 (0.25–0.42)	0.32
Employed part-time	0.25 (0.23–0.27)	0.16 (0.04–0.44)	
Unemployed	0.46 (0.45–0.47)	0.49 (0.42–0.56)	
Other	0.05 (0.01–0.17)	0.02 (0.00–0.14)	
Average monthly household income			
Under R1300	0.13 (0.09–0.17)	0.14 (0.08–0.24)	0.33
R1300–R2000	0.10 (0.07–0.15)	0.08 (0.05–0.11)	
R2001–R3000	0.10 (0.04–0.23)	0.18 (0.11–0.28)	
R3001–R4000	0.22 (0.16–0.29)	0.16 (0.07–0.31)	
Over R4001	0.45 (0.30–0.61)	0.44 (0.42–0.47)	
Parity			
2 or more prior children	0.18 (0.12–0.26)	0.30 (0.18–0.46)	0.10
Less than 2 prior children	0.82 (0.72–0.89)	0.70 (0.59–0.79)

**Table 2:** Attendance to ANC visits

	Unadjusted			Adjusted^a^	
	Intervention N (%) n = 87	Control N (%) n = 90	p-value	Intervention vs control	
				Odds ratio (CI)	p-value
Clinician reported care seeking during pregnancy					
Attended at least 2 ANC visits	85 (97.7)	76 (84.4)	0.002	7.77 (2.06–29.29)	0.01
Attended at least 3 ANC visits	80 (92.0)	59 (65.6)	0.000	5.66 (3.16–10.15)	0.00
Attended at least 4 ANC visits	63 (72.4)	41 (45.6)	0.000	3.21 (1.73–5.98)	0.01
Attended at least 5 ANC visits	36 (41.4)	16 (17.8)	0.001		
Attendance to first ANC prior to 21 weeks	0.70 (0.49–0.85)	0.78 (0.46–0.93)	0.56	0.62 (0.11–3.49)	0.503
Mean number of ANC visits attended	4.4 (4.0–4.7)	3.2 (2.9–3.5)	0.000	3.16 (1.56–6.42)	0.009
Gestational age (in days) at first ANC (median–CI)	123 (128.5)	105 (53.5)	0.223	1.72 (0.37–7.98)	0.407

^a^Adjusted for country of birth

The odds of achieving the primary outcome, completing the continuum of care, and therefore receiving comprehensive care, were 3.2 times higher among intervention participants after adjusting for country of birth (OR = 3.2, 95% CI 1.63–6.31, p = 0.007) (Table 3).

**Table 3:** Proportion of infants receiving immunizations and attending complete continuum of care

	Unadjusted			Adjusted^a^	
	Intervention (proportion with CI) n = 87	Control (proportion with CI) n = 90	p-value	Intervention vs control	
				Odds ratio (CI)	p-value
Birth: BCG	0.99 (0.86–0.99)	0.97 (0.83–0.99)	0.40	3.45 (0.10–122)	0.41
Birth: OPV1	0.97 (0.65–0.99)	0.97 (0.83–0.99)	0.98	1.06 (0.03–35.18)	0.97
6 weeks: OPV2	0.99 (0.86–0.99)	0.98 (0.85–0.99)	0.62	1.05 (0.01–179.47)	0.98
6 weeks: RV1	0.99 (0.86–0.99)	0.98 (0.85–0.99)	0.62	1.05 (0.01–179.47)	0.98
6 weeks: Dtap-IPV-Hib1	0.99 (0.96–0.99)	0.97 (0.93–0.98)	0.12	2.83 (0.69–11.64)	0.12
6 weeks: PCV1	0.99 (0.86–0.99)	0.98 (0.85–0.99)	0.62	1.05 (0.01–179.47)	0.98
6 weeks: Hep B1	0.99 (0.86–0.99)	0.98 (0.85–0.99)	0.62	1.41 (0.06–35.67)	0.80
10 weeks: Dtap-IPV-Hib2	0.98 (0.96–0.99)	0.97 (0.94–0.98)	0.33	–	-
10 weeks: Hep B2	1	0.98 (0.97–0.98)	0.12	1	-
14 weeks: Dtap-IPV-Hib3	1	0.94 (0.91–0.97)	0.12	1	-
14 weeks: Hep B3	0.99 (0.86–0.99)	0.97 (0.78–0.99)	0.43	2.83 (0.08–96.75)	0.76
14 weeks: PCV2	0.98 (0.75–0.99)	0.97 (0.78–0.99)	0.78	1.50 (0.05–45.45)	0.77
14 weeks: RV2	0.97 (0.88–0.99)	0.96 (0.88–0.98)	0.71	1.53 (0.25–9.48)	0.58
9 months: Measles1	1	0.94 (0.82–0.98)	0.22	1	
9 months: PCV3	0.98 (0.96–0.99)	0.94 (0.82–0.98)	0.16	2.38 (0.60–9.42)	0.17
Fully immunized	0.95 (0.91–1)	0.89 (0.82–0.96)	0.11	1.73 (0.54–5.52)	0.282
Attending at least 4 ANC visits and fully immunized up to 1 year (complete continuum of care)					
	0.7 (0.60–0.80)	0.41 (0.31–0.51)	0.00	3.2 (1.63–6.31)	0.007

*BCG* Bacille Calmette-Guèrin (cavvine agains tuberculosis), *OPV* oral polio vaccine, *RV* rotavirus, PCV = pneumococcal conjugate vaccine, *Dtap-IPV-Hib* Diphtheria, tetanus, acellular pertussis (whooping cough), inactivated polio vaccine, haemophilus influenzae type B, *Hep B* hepatitis B

^a^Adjusted for country of birth

Regarding ANC attendance, 72% of intervention participants attended four or more ANC visits, compared to 46% of control participants. Adjusting for country of birth, the odds of attending at least 4 ANC visits was therefore 3.21 (1.73–5.98) higher in the intervention group (p = 0.01) (Table 2). The average number of ANC visits in the intervention arm was higher (μ = 4.4; CI 4.0–4.7) than the control arm (μ = 3.2; CI 2.9–3.5, p < 0.001) (Table 2).

Both intervention and control participants had very high infant vaccination coverage. Looking at each vaccination individually, intervention participants attended 99.6% (1300/1305) of all recommended vaccinations, while the control group attended 91.0% (1336/1350), which was not significantly different (RR: 1.01, 95% CI 1–1.01). In total, 95% (0.91–1.0) of intervention children and 89% (0.82–0.96) of control children were fully immunized, which was also not statistically different (p = 0.11, Table 3).

## Discussion

This study shows that participants who received the SMS intervention had an increased uptake of ANC visits and were 71% more likely than control participants to complete the maternal and infant health continuum of care during pregnancy and their infant's first year of life. This positive effect remained when we controlled for differences in baseline characteristics.

To our knowledge, measuring the impact of maternal mHealth messaging on completing the recommended maternal health continuum of care during pregnancy and throughout the first year of life, including both ANC and EPI, has not been investigated before. Previous maternal mHealth research has regularly focussed on either ANC [37, 38], ANC and early PNC [21], or EPI coverage only [39–43]. Systematic reviews have also looked at ANC visits, PNC visits, or EPI coverage separately [44, 45], but not the complete continuum of care. Encouragingly, there is an ongoing ‘quasi-randomised controlled trial’ (as of 2017) in China using a similar stage-based SMS intervention to support pregnant women and new mothers which has identified both ANC and EPI visit attendance as secondary outcomes [46]. The results of that study could allow for an analysis of the continuum of care as seen above. Thus, the results of the current study provide an important addition to the body of evidence regarding mHealth interventions for maternal and infant health continuum of care and service uptake.

The increased likelihood of attending at least four ANC visits could be explained by the additional maternal health information and psychosocial support provided by the message content generally and the ANC attendance reminder messages specifically. A qualitative study conducting focus groups with MAMA users found high acceptability of the maternal text messages and that users found them to be timely, clear and supportive [47]. A similar study by Lund et al. [48], ‘Wired Mothers’ in Zanzibar, showed a similar effect on ANC attendance with a maternal health SMSes plus an airtime voucher intervention; those results showed increased ANC attendance in the intervention group. In Thailand, a study looking at an SMS-based ANC and EPI visit reminder intervention again showed similar results; women who were part of the SMS intervention arm were more likely to attend their scheduled ANC and EPI visits on time [49]. In a previously published study of the same MAMA intervention as the current study, an HIV-infected cohort receiving maternal health SMSes, including PMTCT support, showed a statistically significant increase in average number of ANC visits, attending at least four ANC visits, and fewer low-birthweight infants, compared to a control cohort [35]. Looking outside mHealth interventions, multiple other intervention studies have been unable to show a statistically significant increase the likelihood of intervention participants attending four or more ANC visits [37, 38].

We found extremely high rates of EPI coverage in the control group, and it is therefore unsurprising that there was no significant increase in the intervention group. A factor that could contribute to high EPI coverage that was found is that proof of vaccination is required to be submitted upon application for a public school in South Africa. A non-significant trend towards increased vaccination attendance in the intervention group compared to the control was also found in an SMS-based vaccination appointment reminder intervention piloted in Guatemala [39]. Conversely, an SMS appointment reminder intervention in Nigeria showed that intervention patients that received their reminder message cut non-attendance to PNC visits in half (21.3% vs 42.8%; p < 0.01) [50]. A further SMS-based pre-post intervention study from Bangladesh looking at EPI coverage [43] found that customised vaccination schedule reminder intervention increased the proportion of full vaccination of intervention patients by 18.8% while the proportion of control participants with complete vaccinations decreased by 10.7% in the same time period (OR: 3.6, 95% CI 1.5–8.9). The Nigerian and Bangladeshi studies are both in line with two previous systematic reviews [40, 41] which both looked at non-mHealth EPI rate studies and both showed that patient reminder interventions, especially those which were phone-based, tended to increase EPI coverage.

A significant body of evidence, including the current study, provide evidence that maternal health SMSes can be a useful public health intervention to increase maternal and infant health care attendance. As Labrique et al. [51] have stated, “[a]s the evidence base continues to be strengthened using both conventional and novel methods of evaluation, the gradual adoption of mHealth into mainstream health systems can be expected” (p 468). The Nigerian and Bangladeshi studies above [40, 41] suggest that maternal mHealth interventions could be even more useful in areas starting with low levels of health service utilisation. Those implementing mHealth projects need to be aware that there is no guarantee that they will work in all circumstances and local context and/or customisation is critical and must be taken into consideration. In our setting the high literacy rate and mobile phone ownership made the intervention accessible to the vast majority of the population [25]. A cost-effectiveness analysis suggests the current intervention may be a cost-effective strategy for boosting ANC and EPI attendance in the South African setting [52]. The South Africa National Department of Health in 2014 decided to roll out an mHealth intervention to all pregnant women countrywide. The intervention is based on MAMA, but the name was changed to MomConnect.

### Study limitations

This study is limited in that it only included individuals already using the (public) healthcare system and only included women whose infants survived through their first year of life. Nonetheless, South Africa has very high levels of maternal and infant health service utilisation; which makes the results applicable to the vast majority of the population of pregnant women. Our study findings should be interpreted considering the low sample size: Only 21.4% of the women who had received the intervention could be traced and within the study, a large number of potential participants could not be included due to missing ANC records; as MAMA had stopped enrolment of participants additional recruitment to the study was not possible once this situation was identified. However, the study team has no reason to believe that this missing data is non-random and therefore do not feel the results are biased by it. It was found that the two study cohorts were not entirely homogeneous, with the control group having a lower proportion of South Africa-born participants. This was controlled for within a data analysis model. Lastly, the study methodology did not allow for monitoring of stillbirths, miscarriages or death of the infant before they reached one year of age; including only live infants might have diluted the results.

## Conclusions

This study shows that women receiving the SMS intervention were more likely to complete the maternal and infant health continuum of care and attend ANC visits more frequently when compared to the control group. Based on these results, there seems to be promising evidence that maternal mHealth interventions can improve attendance to maternal, neonatal and infant health care services in urban settings similar to South Africa. Governments, ministries of health, and public and private health care providers should consider scaling up implementation of evidence-based maternal, neonatal and infant mHealth interventions.

## Data Availability

The datasets used and/or analysed during the current study are available from the corresponding author on reasonable request.

## Appendix: Examples of the maternal health SMSes sent to the intervention cohort.

**Table:**

Timing	Message content
Week 23 of pregnancy	You need 4 clinic visits in pregnancy. A visit may take all day, so get a letter from the clinic for your work. (Doesn't have to say pregnancy)
Week 32 of pregnancy	Going to your clinic appointments is important for you & your baby. Even if you feel healthy, please go. Remember you have a right to ask questions!
Week 35 of pregnancy	Breastmilk is the best food for your baby. It should be his very first food as soon as he's born. Your milk helps protect him from infections
Week 5 postnatal	You should have a Road to Health card for your baby. Keep it safe and take it whenever you take your baby to a clinic, even if you've moved
Week 6 postnatal	Vaccines help prevent your baby catching diseases. Each vaccine needs to be given at the right time. The first ones are due at 6 weeks
Week 11 postnatal—HIV message	If your baby tested HIV + at the 6-week test, make sure you get him to a clinic for treatment. If treated early, he will do very well

## Abbreviations

ANC: Antenatal care
EPI: Expanded Programme on Immunization
PNC: Postnatal care
PMTCT: Prevention of mother to child transmission of HIV
RTH: Road to Health
SMS: Short messaging service
mHealth: Mobile health
